# Efficacy and Safety of CAR-Modified T Cell Therapy in Patients with Relapsed or Refractory Multiple Myeloma: A Meta-Analysis of Prospective Clinical Trials

**DOI:** 10.3389/fphar.2020.544754

**Published:** 2020-12-03

**Authors:** Xinrong Xiang, Qiao He, Yang Ou, Wen Wang, Yu Wu

**Affiliations:** ^1^ Hematology Research Laboratory, West China Hospital, Department of Hematology, Sichuan University, Chengdu, China; ^2^ Chinese Evidence-based Medicine Center and Cochrane China Center, West China Hospital, Sichuan University, Chengdu, China

**Keywords:** chimeric antigen receptor, cancer immunotherapy, multiple myeloma, efficacy, safety, meta-analysis

## Abstract

**Background**: In recent years, chimeric antigen receptor-modified T (CAR-T) cell therapy for B-cell leukemia and lymphoma has shown high clinical efficacy. Similar CAR-T clinical trials have also been carried out in patients with refractory/relapsed multiple myeloma (RRMM). However, no systematic review has evaluated the efficacy and safety of CAR-T cell therapy in RRMM. The purpose of this study was to fill this literature gap.

**Methods**: Eligible studies were searched in PUBMED, EMBASE, the Cochrane Central Register of Controlled Trials (CENTRAL), CNKI, and WanFang from data inception to December 2019. For efficacy assessment, the overall response rate (ORR), minimal residual disease (MRD) negativity rate, strict complete response (sCR), complete response (CR), very good partial response (VGPR), and partial response (PR) were calculated. The incidence of any grade cytokine release syndrome (CRS) and grade ≥3 adverse events (AEs) were calculated for safety analysis. The effect estimates were then pooled using an inverse variance method.

**Results**: Overall, 27 studies involving 497 patients were included in this meta-analysis. The pooled ORR and MRD negativity rate were 89% (95% Cl: 83–94%) and 81% (95% Cl: 67–91%), respectively. The pooled sCR, CR, VGPR, and PR were 14% (95% Cl: 5–27%), 13% (95% Cl: 4–26%), 23% (95% Cl: 14–33%), and 15% (95% Cl: 10–21%), respectively. Subgroup analyses of ORR by age, proportion of previous autologous stem cell transplantation (ASCT), and target selection of CAR-T cells revealed that age ≤ 55 years (≤55 years vs. > 55 years, *p* = 0.0081), prior ASCT ≤70% (≤70% vs. > 70%, *p* = 0.035), and bispecific CAR-T cells (dual B-cell maturation antigen (BCMA)/BCMA + CD19 vs specific BCMA, *p* = 0.0329) associated with higher ORR in patients. Subgroup analyses of remission depth by target selection suggested that more patients achieved a better response than VGPR with dual BCMA/BCMA + CD19 CAR-T cells compared to specific BCMA targeting (*p* = 0.0061). In terms of safety, the pooled incidence of any grade and grade ≥ 3 CRS was 76% (95% CL: 63–87%) and 11% (95% CL: 6–17%). The most common grade ≥ 3 AEs were hematologic toxic effects.

**Conclusion**: In heavily treated patients, CAR-T therapy associates with promising responses and tolerable AEs, as well as CRS in RRMM. However, additional information regarding the durability of CAR-T cell therapy, as well as further randomized controlled trials, is needed.

## 1. Introduction

Multiple myeloma (MM) is the second most common hematological malignancy after non-Hodgkin’s lymphoma. It is characterized by clonal evolution of malignant plasma cells (Lipe et al., 2016). During the past decades, autologous stem cell transplantation (ASCT) and the development of novel agents, such as proteasome inhibitors (PIs), immunomodulatory drugs (IMiDs), and monoclonal antibodies, have significantly prolonged patient survival. Although MM treatment options have gradually improved, relapsed and refractory diseases are common (Palumbo and Anderson, 2011; Rajkumar, 2011; Chim et al., 2018; Goldschmidt et al., 2019). It is, therefore necessary to develop innovative treatment strategies to achieve long-term remission for patients with relapsed/refractory MM.

Chimeric antigen receptor (CAR)-T cell therapy has shown the potential for inducing durable remission in certain hematologic malignancies (Makita et al., 2017; Mikkilineni and Kochenderfer, 2017; Neelapu et al., 2018). Meanwhile, anti-CD19 CAR-T-cell therapies reportedly offer promising efficacy in patients with leukemia or lymphoma. Based on previous successful results in B-cell neoplasms (Maude et al., 2014; Lee et al., 2015; Turtle et al., 2016a; Kochenderfer et al., 2017; Neelapu et al., 2017; Jain et al., 2018; Maude et al., 2018; Park et al., 2018), this approach has been licensed by the US Food and Drug Administration (FDA) for the treatment of relapsed or refractory acute lymphocytic leukemia (ALL), and diffuse large B-cell lymphoma (DLBCL). CAR-T cell therapy is defined as a novel immunotherapy that modifies T-cells with CAR, typically consisting of a target-recognition ectodomain, an anchored functional transmembrane domain, a hinge region, and signaling endodomains (Jensen and Riddell, 2015; van der Stegen et al., 2015; Guedan et al., 2018). Selection of targets is the key to successful CAR-T therapy (Melchor et al., 2014). Currently, in the context of RRMM, targets used in clinical trials include the B-cell maturation antigen (BCMA), CD19, CD138, signaling lymphocytic activation molecule 7 (SLAM7), immunoglobulin light chains, and the fully human heavy-chain variable domain (FHVH) (Hajek et al., 2013; Lam et al., 2020).

Design and optimization of CAR-T therapy in RRMM has been a hot research area with several prospective clinical trials having been conducted to evaluate its efficacy and safety. However, there is a lack of quantitative and comprehensive statistical analyses on treatment outcome. Moreover, the factors contributing to CAR-T-cell therapy efficacy and safety in RRMM patients remain unclear. Therefore, a systematic review and meta-analysis on the efficacy and safety of the CAR-modified T cell therapy in RRMM patients were performed to offer an evidence-based reference for clinicians.

## 2. Materials and Methods

### 2.1. Methods

In performing this study, we abided by the standards set by the Preferred Reporting Items for Systematic Reviews and Meta-Analyses (PRISMA) (Knobloch et al., 2011).

### 2.2. Literature Search

We searched PUBMED, EMBASE, the Cochrane Central Register of Controlled Trials (CENTRAL), CNKI, and WanFang from inception of the study to December 20, 2019 without any language restriction. We combined Medical Subject Headings (MeSH) terms and free-text terms regarding “CAR” and “myeloma” to search for potentially eligible studies.

### 2.3. Inclusion and Exclusion Criteria

We included clinical trials (phase 1 and phase 2 single arm trials) involving patients with relapsed or refractory MM receiving CAR-T cell therapy. Qualified studies reported at least one of the following variables: efficacy outcomes (overall response rate, ORR), strict complete response (sCR), complete response (CR), very good partial response (VGPR), partial response (PR), minimal residual disease (MRD) negativity rate, and safety outcomes (any grade cytokine syndrome, CRS), grade ≥ 3 AEs (anemia, neutropenia, lymphopenia, thrombocytopenia), and grade ≥ 3 CAR-T- related encephalopathy syndrome (CRES). No restrictions on sample size or length of follow-up were imposed.

### 2.4. Study Qualitative Assessment

The Methodological Index for Non-randomized Studies (MINORS) was adopted to assess the methodological quality of the inclusive studies. MINORS contained 12 items, eight of which were specified for non-comparative studies (Slim et al., 2003; Cullis et al., 2020). The eight items included: study aims, consecutive patient inclusion criteria, prospective pooling of data, endpoint consistent with the study aim, unbiased evaluation of endpoints, follow-up period, loss to follow-up less than 5%, and prospective calculation of the sample size. The items were scored 0 (not reported), 1 (reported but inadequate), or 2 (reported and adequate).

### 2.5. Data Extraction

Two investigators independently reviewed and extracted the following information: study characteristics (first author, publication year, ClinicalTrials.gov number, research design), patient characteristics (the group number, age, median time from diagnosis, prior lines of treatment, high-risk cytogenetics, previous ASCT, anti-CD38 monoclonal antibodies exposed, extramedullary-disease), intervention (CAR-T cell dose, target selection, costimulatory domain, conditioning regimen), and outcomes of interest (treatment response, adverse events (AEs)). Discrepancies were settled by discussion or by adjudication by a third reviewer.

### 2.6. Statistical Analysis

We used the Metaprop module in the R-3.4.3 statistical software package to analyze therapeutic efficacy and safety. The effect estimates were pooled using an inverse variance method. Heterogeneity among studies was evaluated by the chi-squared test (*χ*2 test) and I-squared test (*I* ^2^ test). In case of potential heterogeneity (*I* ^2^ > 50%), analysis was conducted using the random-effect model; otherwise, the fixed-effect model was employed. Subgroup analysis by age (≤55 vs. >55 years), proportion of high-risk cytogenetics (≤50% vs. >50%), proportion of previous ASCT (≤70% vs. >70%), conditioning regimen (cyclophosphamide plus fludarabine vs cyclophosphamide only), target selection for CAR-T therapy (specific BCMA vs. dual BCMA/BCMA + CD19 vs BCMA + others), costimulatory domain (4-1BB vs. CD28 vs. CD28 + OX40) was performed to explore the sources of heterogeneity. P values < 0.05 were considered statistically significant. Sensitivity analysis was aimed at estimating the effect with removal of the largest sample size among all studies.

## 3. Results

### 3.1. Literature Search Results and Study Characteristics

**FIGURE 1:**
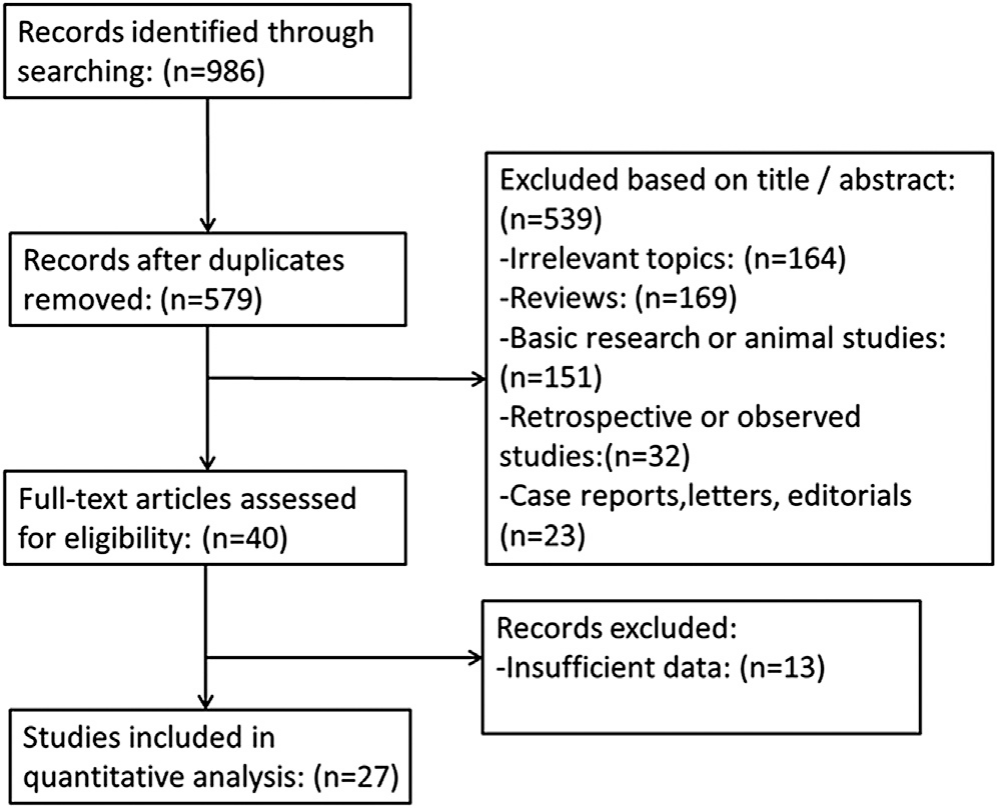
Flow diagram of the study selection process.

The flowchart illustrating the literature search process is presented in Figure 1. Our search yielded 986 reports, 407 of which were, duplicates. After screening titles, abstracts, and full text, 552 publications were excluded. Ultimately, 27 studies, involving 497 patients, were included (Fan et al., 2017; Yan et al., 2017; Zhang et al., 2017; Brudno et al., 2018; Zhao et al., 2018; Berdeja et al., 2019; Chen et al., 2019; Cohen et al., 2019; Costello et al., 2019; Cowan et al., 2019; Fu et al., 2019; Han et al., 2019; Jie et al., 2019; Li et al., 2019a; Li et al., 2019b; Madduri et al., 2019; Mikkilineni et al., 2019; Popat et al., 2019; Raje et al., 2019; Yan et al., 2019) (Mailankody et al., 2018a; Mailankody et al., 2018b; Damian et al., 2018; Han et al., 2018; Jiang et al., 2018; Li et al., 2018; Shi et al., 2018).

Table 1 shows the characteristics of the inclusive studies. All studies were single-arm clinical trials, and involved 497 patients who had received at least two lines of treatment. Of the 27 included studies, 17 (63%) explored the efficacy and safety of the specific BCMA CAR-T therapy in patients with RRMM (Zhang et al., 2017; Brudno et al., 2018; Cohen et al., 2019; Costello et al., 2019; Cowan et al., 2019; Fu et al., 2019; Jie et al., 2019; Li et al., 2019b; Madduri et al., 2019; Popat et al., 2019; Raje et al., 2019) (Damian et al., 2018; Han et al., 2018; Jiang et al., 2018; Li et al., 2018; Mailankody et al., 2018a; Mailankody et al., 2018b), four (15%) focused on targeting of the dual BCMA (Fan et al., 2017; Zhang et al., 2017; Zhao et al., 2018; Chen et al., 2019), three (11%) explored the targeting of BCMA plus CD19 (Yan et al., 2017; Yan et al., 2019) (Shi et al., 2018), and the remaining three (11%) examined the targeting of BCMA plus other targets, i.e., CD38, FHVH, and the transmembrane activator and calcium-modulator and cyclophilin ligand interactor (TACI), respectively (Li et al., 2019a; Mikkilineni et al., 2019; Popat et al., 2019). The CAR-T cell dose varied across studies and ranged between 0.07 × 10^6^ and 82 × 10^6^ cells/kg. The costimulatory domain was either 4-1BB or CD28. For conditioning regimen, the common choices were cyclophosphamide (CP) alone or in combination with fludarabine (Flu). The mean patient age ranged from 53 to 64.5 years; the median time from diagnosis was 3.5–5 years; the proportion of anti-CD38 mAb exposure was 20.80–100%; the proportion of prior ASCT was 18.20–97%; the proportion of extramedullary-disease was 18.75–45.80%; and the proportion of high-risk patients was 32–100% (Table 2).

**TABLE 1:** Characteristics of the included studies.

No	Study	Registration number	No. of patients	Design	Target	Treatment	Costimulatory domain	Conditioning
1	Raje et al. (2019)	NCT02658929	33	Phase1, single arm	BCMA (bb2121)	150/450/800 × 10^6 cells	4-1BB	CP/Flu
2	Brudno et al. (2018)	NCT02215967	16	Phase I, single arm	BCMA	9 × 10^6 cells/kg	CD28	CP/Flu
3	Fan et al. (2017)	—	19	Phase I/II, single arm	LCAR-B38M	4.7 (0.6–7.0) x 10^6/kg	4-1BB	CP
4	Zhang et al. (2017)	—	22	Phase I, single arm	LCAR-B38M	4.0 × 10^6 (1.5–7.0 × 10^6)/kg	4-1BB	CP
5	Hao et al. (2019)	NCT03716856, NCT03302403, NCT03380039	24	3-Site phase I, single arm	BCMA（CT053)	1.5 × 10^8 cells	4-1BB	CP/Flu
6	Han et al. (2019)	—	16	Phase I, single arm	BCMA	2–10 × 10^6 cells/kg	4-1BB	CP/Flu
7	Shah et al. (2020)	NCT03274219	22	Multicenter phase I, single arm	BCMA(bb21217)	150/300/450 × 10^6 cells	4-1BB	CP/Flu
8	Zhao et al. (2018)	NCT03090659	57	Multisite phase1/2	LCAR-B38M	0.07–2.1 × 10^6 cells/kg	4-1BB	CP
9	Jie et al. (2019)	ChiCTR—ONH—17012285	17	Multisite phase1/2, single arm	LCAR-B38M	0.21–1.52 × 10^6 cells/kg	4-1BB	CP/Flu or CP only
10	Gregory et al. (2018)	NCT03288493	12	Phase I, single arm	BCMA	0.75–15 × 10^6 cells	4-1BB	CP/Flu
11	Mailankody et al. (2018a)	NCT03430011	19	Multisite phase1/2, single arm	BCMA	50/150 × 10^6 cells (5 + 3)	4-1BB	CP/Flu
12	Jiang et al. (2018)	NCT03915184	16	Multisite phase1(CT053)	BCMA (CT053), single arm	0.5/1.5/1.8 × 10^8 cells	4-1BB	CP/Flu
13	Mailankody et al. (2018b)	NCT03070327	11	Phase 1 (MCARH171), single arm	BCMA	72/137/475/818 × 10^6 cells	4-1BB	CP/Flu
14	Li et al. (2018)	ChiCTR—OPC—16009113	28	Phase 1 (BRD015), single arm	BCMA	5.4–25.0 × 10^6 cells/kg	CD28	CP/Flu
15	Li et al. (2019a)	ChiCTR1800018137	16	Phase 1 (CT103A), single arm	BCMA	1/3/6/8 × 10^6 cells/kg	4-1BB	CP/Flu
16	Cohen et al. (2019)	NCT02546167	25	Single arm phase 1	BCMA	1–5 × 10^7/10^8 cells	4-1BB	CP or none
17	Fu et al. (2019)	NCT03093168	44	Single arm phase 1	BCMA	9 × 10^6 cells/kg	4-1BB	CP/Flu
18	Han et al. (2018)	NCT03661554	4	Multisite phase 1; single arm	BCMA	5/10 × 10^6 cells/kg	4-1BB	CP/Flu
19	Yan et al. (2017)	NCT03196414	8	Single arm	BCMA + CD19	1 × 10^7/kg CD19-targeted cells; 2.5–8.2 × 10^7/kg BCMA-targeted cells	OX40, CD28	CP/Flu
20	Shi et al. (2018)	NCT03455972	9	Single arm	BCMA + CD19	1 × 10^7/kg CD19-targeted cells; 2.5–8.2 × 10^7/kg BCMA-targeted cells	OX40, CD28	BUCY + ASCT
21	Yan et al. (2019)	ChiCTR—OIC—17011272	21	Single arm, phase 2 trial	BCMA + CD19	1 × 10^6/kg both BCMA and CD19-targeted CAR + T cells	4-1BB	CP/Flu
22	Damian (2018)	NCT03338972	7	Phase I, single arm	BCMA	5–15× 10^7 cells	4-1BB	Null
23	Cowan et al. (2019)	NCT03502577	6	Phase I single arm, with an orally administered gamma secretase inhibitor (JSMD194)	BCMA？	5 × 10^7 EGFRt + cells	4-1BB	Null
24	Madduri et al. (2019)	NCT03548207	25	Phase 1b/2 single arm study of JNJ-4528 (containing two BCMA targeting)	BCMA	0.75 × 10^6 cells/kg (0.5–1.0 × 10^6)	Null	CP/Flu
25	Li et al. (2019b)	ChiCTR1800018143	16	Phase 1 single arm (BM38)	BCMA + CD38	0.5/1.0/2.0/3.0/4.0 × 10^6 cells/kg	4-1BB	CP/Flu
26	Popat et al. (2019)	—	12	Phase 1 first-in-human study of AUTO2, single arm	BCMA + TACI	15/75/225/600/900 × 10^6 cells	CD28	CP/Flu
27	Mikkilineni et al. (2019)	—	12	Single arm	FHVH-BCMA-T	0.75/1.5/3 × 10^6 cells/kg	4-1BB	CP/Flu

BCMA,B-cell maturation antigen; FHVH, fully human heavy-chain variable domain; LCAR-B38M, bispecific BCMA; TACI, transmembrane activator and calcium-modulator and cyclophilin ligand interactor; CP,cyclophosphamide; Flu,fludarabine; mAb, monoclonal antibody; ASCT, autologous stem cell transplant.

**TABLE 2:** Characteristics of the included patients.

No	Study	No. of patients	Mean age (years)	prior lines	median time from diagnosis, (years)	High-risk-cytogenetics (%)	Prior ASCT (%)	Anti-CD38 mAb exposed (%)	Extramedullary-disease (%)
1	Raje et al. (2019)	33	60	7	5	45.00%	97.00%	79.00%	27.00%
2	Brudno et al. (2018)	16	—	9.5	—	40.00%	75.00%	43.75%	—
3	Fan et al. (2017)	19	—	—	—	—	—	—	—
4	Zhang et al. (2017)	22	53.5	—	—	—	18.20%	—	—
5	Hao et al. (2019)	24	60.1	4.5	3.5	37.50%	41.70%	20.80%	45.80%
6	Han et al. (2019)	16	—	10	—	—	—	—	18.75%
7	Shah et al. (2020)	22	63	7	—	31.82%	82.00%	86.00%	—
8	Zhao et al. (2018)	57	54	3	4	—	58.00%	0.00%	—
9	Jie et al. (2019)	17	56	4	—	—	47.05%	—	29.41%
10	Gregory et al. (2018)	12	—	—	—	64.00%	—	100.00%	—
11	Mailankody et al. (2018a)	19	53	10	4	50.00%	88.00%	—	—
12	Jiang et al. (2018)	16	55	4	3.9	—	56.00%	—	—
13	Mailankody et al. (2018b)	11	—	6	—	82.00%	—	100.00%	—
14	Li et al. (2018)	28	—	—	—	—	—	—	—
15	Li et al. (2019a)	16	—	—	—	—	—	—	—
16	Cohen et al. (2019)	25	58	7	4.6	96.00%	92.00%	76.00%	28.00%
17	Fu et al. (2019)	44	—	—	—	—	—	—	—
18	Han et al. (2018)	4	57	—	—	—	—	—	—
19	Yan et al. (2017)	8	—	4	—	—	—	—	—
20	Shi et al. (2018)	9	55	—	—	—	—	—	—
21	Yan et al. (2019)	21	—	—	—	—	—	—	—
22	Damian (2018)	7	63	8	—	100.00%	71.00%	—	—
23	Cowan et al. (2019)	6	64.5	10	—	75.00%	—	—	—
24	Madduri et al. (2019)	25	61	5	—	—	—	100.00%	—
25	Li et al. (2019b)	16	61	—	—	—	—	—	31.25%
26	Popat et al. (2019)	12	61	5	—	—	73.00%	—	—
27	Mikkilineni et al. (2019)	12	63	6	—	—	—	—	—

BCMA,B-cell maturation antigen; FHVH, fully human heavy-chain variable domain; LCAR-B38M, bispecific BCMA; TACI, transmembrane activator and calcium-modulator and cyclophilin ligand interactor; CP,cyclophosphamide; Flu,fludarabine; mAb, monoclonal antibody; ASCT, autologous stem cell transplant.

### 3.2. Study Quality

All studies illustrated the aim of the study. Their endpoint was appropriate to the aim of the study and data were prospectively collected. In most studies (approximately 80%) consecutive patients were enrolled, an unbiased evaluation of endpoints was performed, and loss to follow-up did not exceed 5%. Twenty-six studies (96%) did not prospectively calculate the sample size. In general, the overall rating was high, and the overall quality of the selected studies was adequate (Table 3).

**TABLE 3:** The scores of MINORS.

Study	1	2	3	4	5	6	7	8	Total
Raje et al. (2019)	2	2	2	2	2	2	2	0	14
Brudno et al. (2018)	2	2	2	2	2	2	2	2	16
Fan et al. (2017)	2	2	2	2	2	2	2	0	14
Zhang et al. (2017)	2	2	2	2	2	2	2	0	14
Hao et al. (2019)	2	2	2	2	2	0	2	0	12
Han et al. 2019	2	2	2	2	2	2	2	0	14
Shah et al. (2020)	2	2	2	2	2	2	2	0	14
Zhao et al. (2018)	2	2	2	2	2	2	2	0	14
Jie et al. (2019)	2	2	2	2	2	2	2	0	14
Gregory et al. (2018)	2	2	2	2	2	2	0	0	12
Mailankody et al. (2018)	2	0	2	2	0	0	0	0	6
Jiang et al. (2018)	2	0	2	2	2	0	0	0	8
Mailankody et al. (2018)	2	0	2	2	2	0	2	0	10
Li et al. (2018)	2	0	2	2	0	0	2	0	8
Li et al. (2019a)	2	2	2	2	2	2	2	0	14
Cohen et al. (2019)	2	2	2	2	2	2	2	0	14
Fu et al. (2019)	2	2	2	2	2	2	2	0	14
Han et al. (2018)	2	0	2	2	2	2	2	0	12
Yan et al. (2017)	2	2	2	2	2	2	0	0	12
Shi et al. (2018)	2	0	2	2	2	0	2	0	10
Yan et al. (2019)	2	2	2	2	2	2	2	0	14
Damian (2018)	2	0	2	2	2	0	2	0	10
Cowan et al. (2019)	2	2	2	2	2	2	2	0	14
Madduri et al. (2019)	2	2	2	2	2	2	2	0	14
Li et al. (2019b)	2	2	2	2	2	2	2	0	14
Popat et al. (2019)	2	2	2	2	2	2	2	0	14
Mikkilineni et al. (2019)	2	2	2	2	0	2	2	0	12

### 3.3. Efficacy of the CAR-Modified T Cell Therapy

**FIGURE 2:**
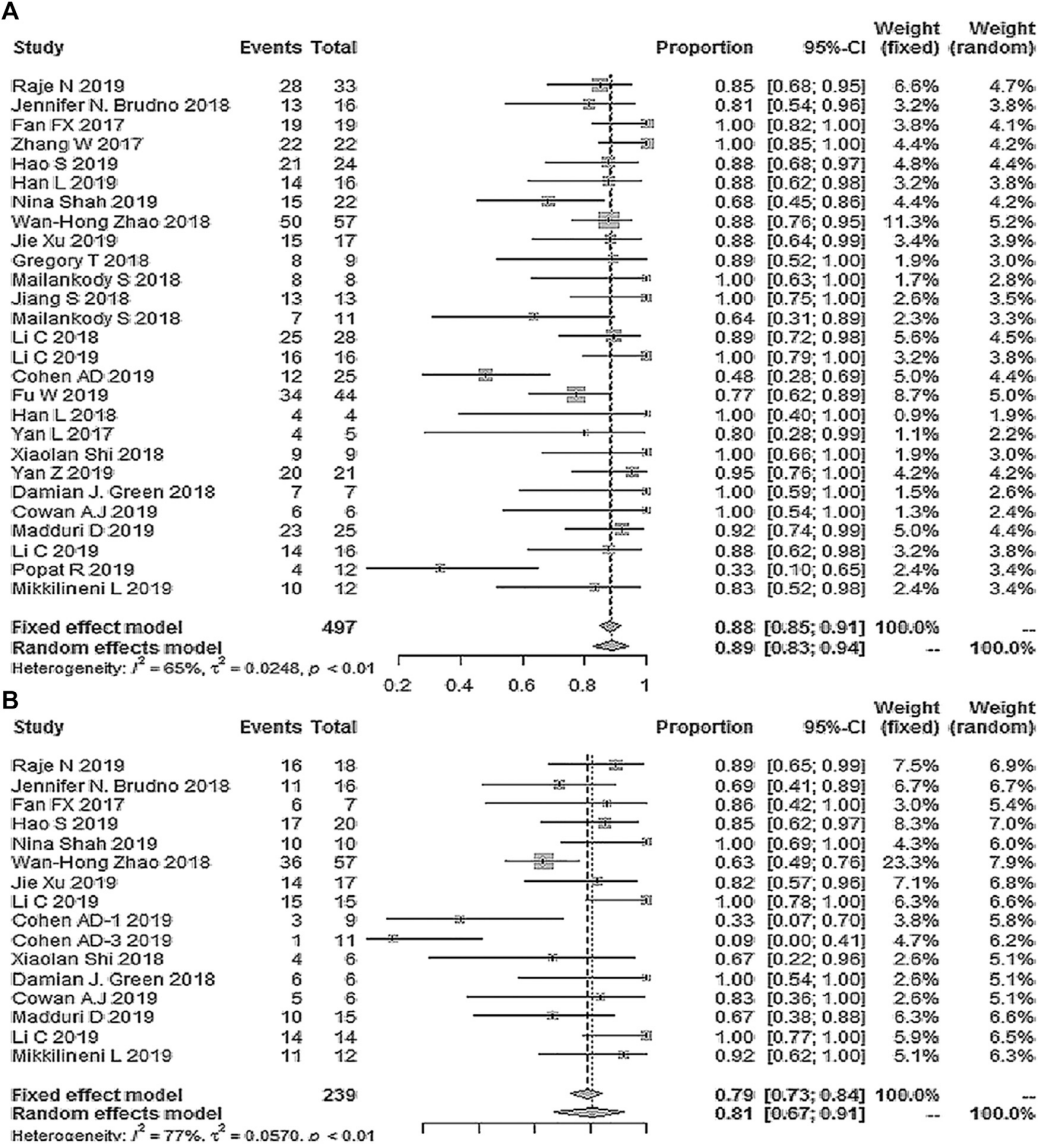
The forest plot of **(A)** pooled ORR, and **(B)**, MRD negativity in patients who received CAR-T cell therapy.

**FIGURE 3:**
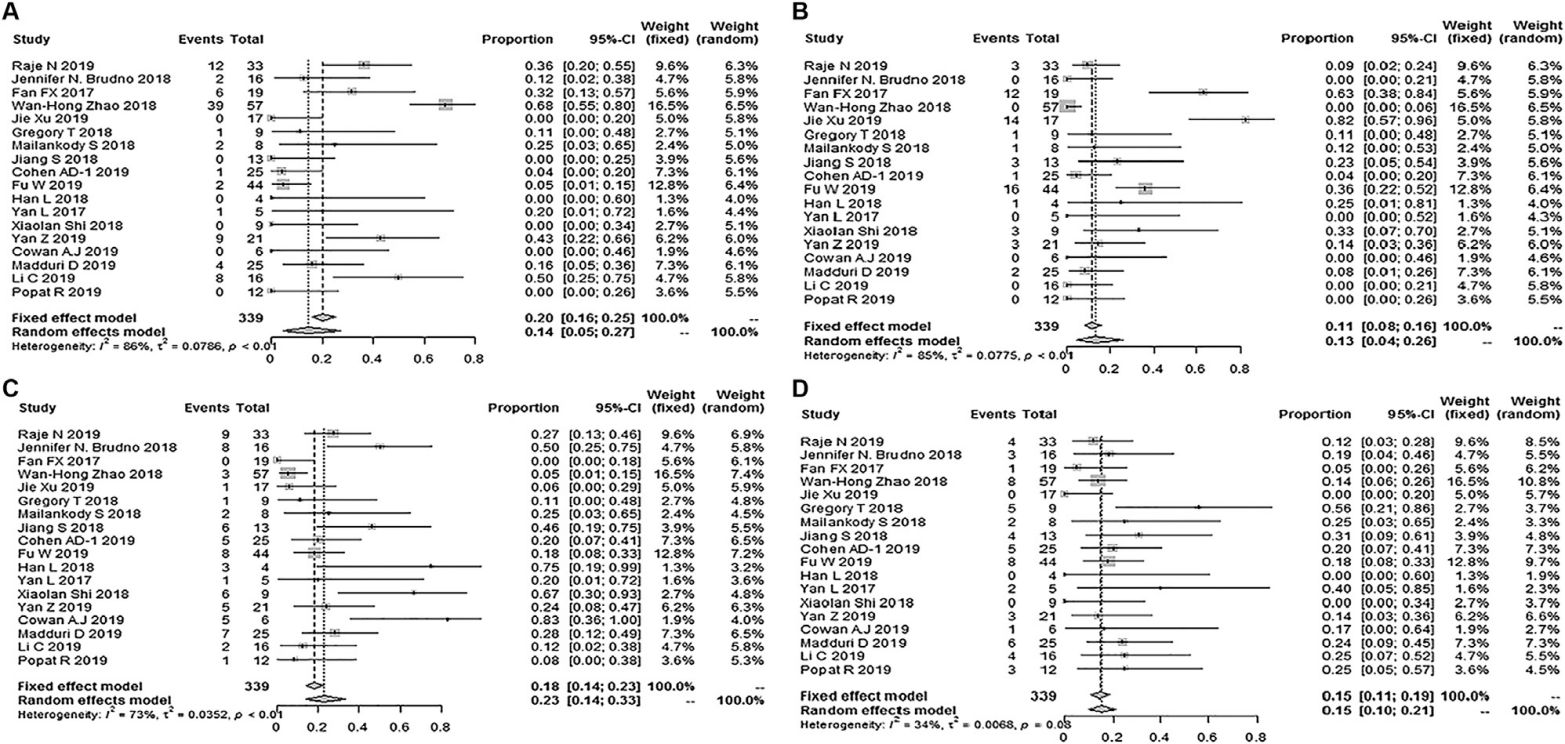
The forest plot of **(A)** pooled sCR, **(B)** CR, **(C)**, VGPR, and **(D)** PR.

Twenty-seven studies with 497 patients reported ORR; the pooled ORR was 89% (95% Cl: 83–94%; Figure 2). Fifteen studies reported the minimal residual disease status, and the pooled MRD negativity rate was 81% (95% Cl: 67–91%) among 239 patients who responded to CAR-T therapy (Figure 2) (Fan et al., 2017; Brudno et al., 2018; Zhao et al., 2018; Berdeja et al., 2019; Chen et al., 2019; Cohen et al., 2019; Jie et al., 2019; Li et al., 2019a; Li et al., 2019b; Madduri et al., 2019; Mikkilineni et al., 2019; Raje et al., 2019) (Damian et al., 2018; Jiang et al., 2018; Shi et al., 2018). Eighteen studies with 339 patients reported the response depth (sCR, CR, VGPR, PR) (Fan et al., 2017; Zhang et al., 2017; Brudno et al., 2018; Berdeja et al., 2019; Chen et al., 2019; Cohen et al., 2019; Jie et al., 2019; Li et al., 2019a; Li et al., 2019b; Madduri et al., 2019; Mikkilineni et al., 2019; Raje et al., 2019) (Damian et al., 2018; Jiang et al., 2018; Shi et al., 2018). The pooled sCR, CR, VGPR, and PR were 14% (95% Cl: 5–27%), 13% (95% Cl: 4–26%), 23% (95% Cl: 14–33%), and 15% (95% Cl: 10–21%), respectively (Figure 3).

**FIGURE 4:**
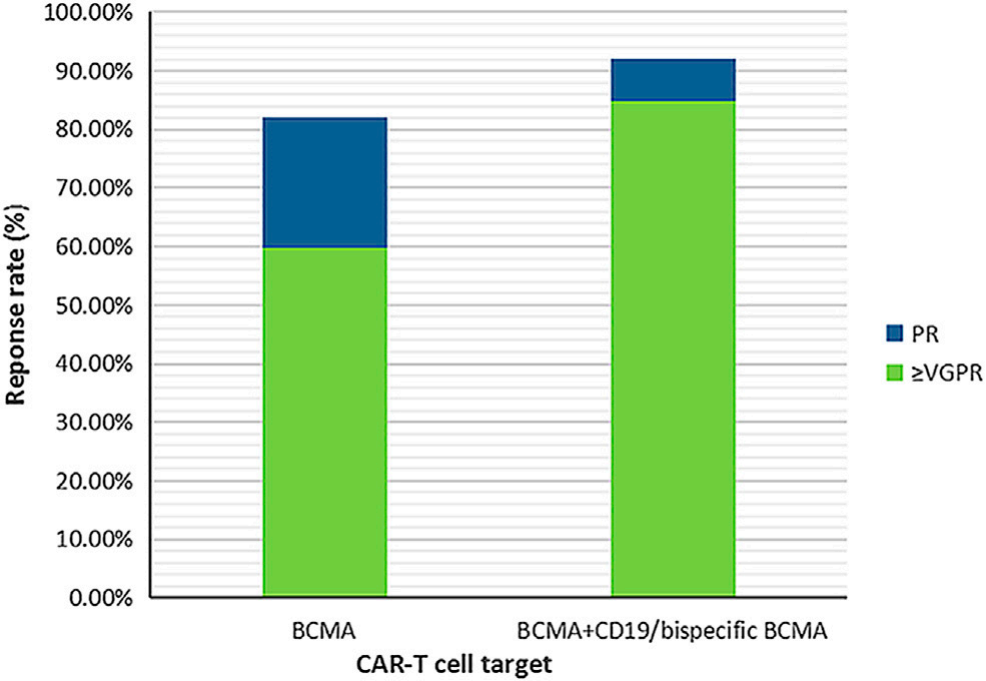
The remission depth achieved by different target selection.

Subgroup analysis of ORR by age showed that, in patients with mean age ≤55 years, the ORR was higher than in those with >55 years (98.01% vs. 82.58%, interaction *p* = 0.0081). Compared to the proportion of prior ASCT > 70%, a higher ORR was observed with a higher proportion of prior ASCT ≤ 70% (93.68% vs. 76.12%, interaction *p* = 0.035). Regarding target selection, the ORR obtained by targeting dual BCMA or BCMA + CD19 was higher than that obtained by targeting specific BCMA or BCMA plus other antigens (96.05% vs. 86.18% vs. 70.28%, interaction *p* = 0.0329). However, subgroup analysis of ORR suggested that no significant differences occurred in the proportion of high-risk cytogenetics patients (≤50% vs. >50%), the use of different costimulatory domains (4-1BB vs CD28 vs CD28 + OX40), or in patients pretreated with CP in the presence or absence of Flu (Table 4). Subgroup analysis of remission depth (sCR, CR, VGPR, PR) suggested that compared to targeting specific BCMA, a higher proportion of patients achieved a better response than VGPR in the case of dual BCMA or BCMA + CD19 targeting (59.89% vs. 84.82%, interaction p = 0.0061). These results are shown in Figure 4 and Table 5.

**TABLE 4:** Subgroup analysis results of ORR.

Subgroup	No of trails	ORR (95% CI)	*p* for differences
**Age,y**			
≤55	5	0.9801 [0.9099; 1.00]	
>55	12	0.8258 [0.7093; 0.9211]	0.0081
**High-risk cytogenetics (%)**			
≤50%	5	0.8421 [0.7421; 0.9237]	
>50%	5	0.8217 [0.5556; 0.9909]	0.7841
**Previous ASCT, rate (%)**			
≤70%	5	0.9368 [0.8584; 0.9887]	
>70%	7	0.7612 [0.5685; 0.9153]	0.035
**Condition regimen**			
CP	5	0.8632 [0.6256; 0.9981]	
CP/Flu	19	0.8680 [0.8013; 0.9247]	0.9628
**CAR-T target**			
BCMA	19	0.8618 [0.7842; 0.9269]	
BCMA + CD19/bispecific BCMA	7	0.9605 [0.8964; 0.9979]	
BCMA + others	3	0.7028 [0.3483; 0.9649]	0.0329
BCMA	19	0.8618 [0.7842; 0.9269]	
BCMA + CD19/bispecific BCMA	7	0.9605 [0.8964; 0.9979]	0.0254
**Costimulatory domain**			
4-1BB	21	0.9024 [0.8382; 0.9542]	
CD28	3	0.7149 [0.3723; 0.9642]	
OX40, CD28	2	0.9559 [0.6435; 1.0000]	0.4385

**TABLE 5:** The subgroup analysis results of response depth.

Subgroup	No of trails	sCR + CR + VGPR (95% CI)	*p* for differences
CAR-T target			
BCMA	10	0.5989 [0.4732; 0.7192]	
BCMA + CD19/bispecific BCMA	6	0.8482 [0.7161; 0.9491]	0.0061
**Subgroup**	**No of trails**	**PR (95% CI)**	***p* For differences**
CAR-T target			
BCMA	10	0.2228 [0.1380; 0.3186]	
BCMA + CD19/bispecific BCMA	6	0.0733 [0.0115; 0.1661]	0.0162

### 3.4. Safety of the CAR-Modified T Cell Therapy

**FIGURE 5:**
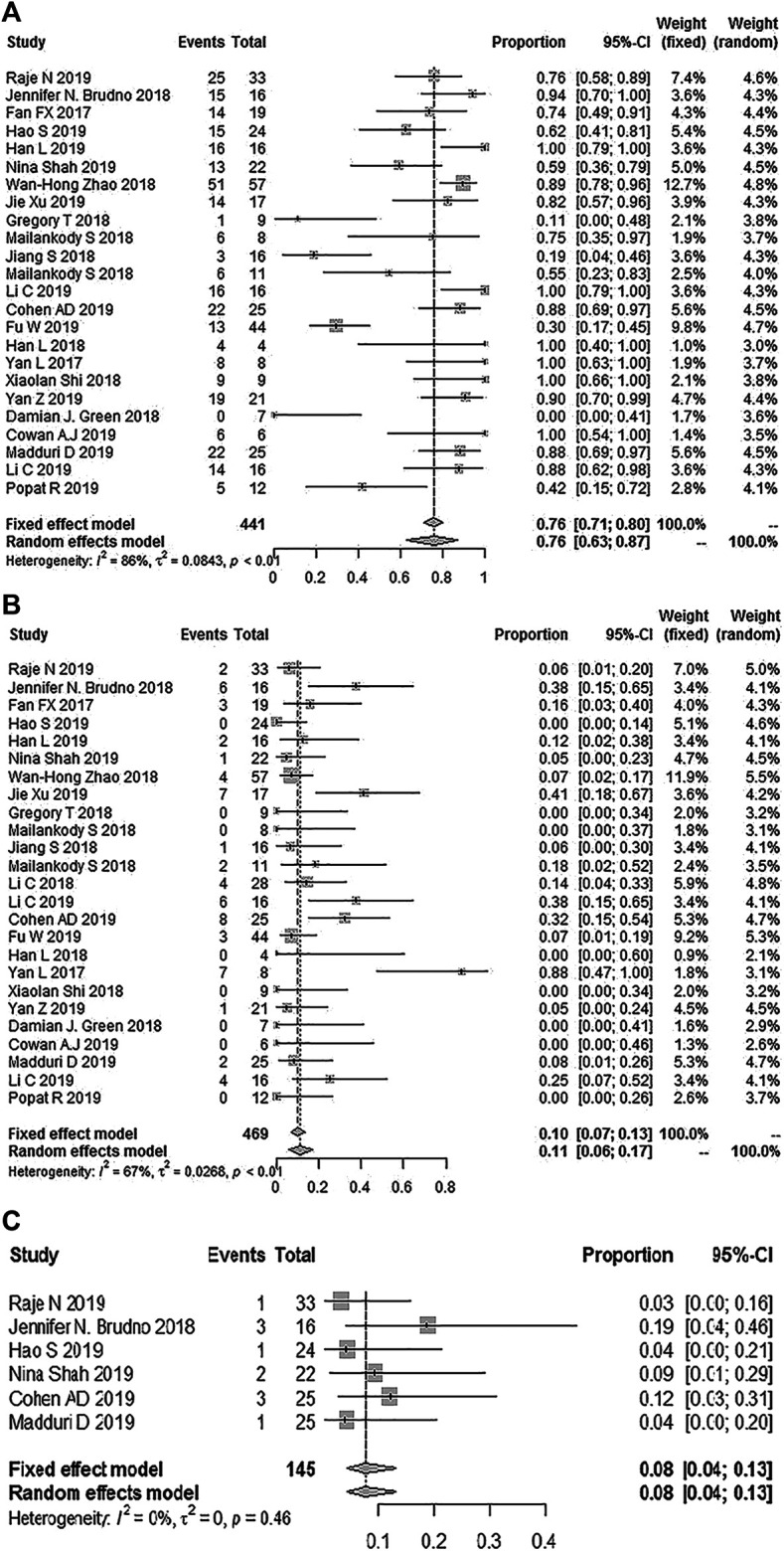
The forest plot of pooled incidence of **(A)** all grade CRS **(B)**, CRS grade ≥3 and **(C)** CRES grade ≥3.

Twenty-four studies reported any grade CRS, and the total incidence of any grade CRS was 76% (95% CL: 63–87%) (Fan et al., 2017; Yan et al., 2017; Brudno et al., 2018; Zhao et al., 2018; Berdeja et al., 2019; Chen et al., 2019; Cohen et al., 2019; Costello et al., 2019; Cowan et al., 2019; Fu et al., 2019; Han et al., 2019; Jie et al., 2019; Li et al., 2019a; Li et al., 2019b; Madduri et al., 2019; Popat et al., 2019; Raje et al., 2019; Yan et al., 2019; Shah et al., 2020) (Damian et al., 2018; Han et al., 2018; Jiang et al., 2018; Mailankody et al., 2018a; Mailankody et al., 2018b; Shi et al., 2018). Twenty-five studies reported grade ≥3 CRS, and the pooled incidence of grade ≥ 3 CRS was 11% (95% CL: 6–17%) (Fan et al., 2017; Yan et al., 2017; Brudno et al., 2018; Zhao et al., 2018; Berdeja et al., 2019; Chen et al., 2019; Cohen et al., 2019; Costello et al., 2019; Cowan et al., 2019; Fu et al., 2019; Han et al., 2019; Jie et al., 2019; Li et al., 2019a; Li et al., 2019b; Madduri et al., 2019; Popat et al., 2019; Raje et al., 2019; Yan et al., 2019; Shah et al., 2020) (Damian et al., 2018; Han et al., 2018; Jiang et al., 2018; Li et al., 2018; Mailankody et al., 2018a; Mailankody et al., 2018b; Shi et al., 2018). Six studies reported a severe CRES, and the relevant pooled incidence was 8% (95% CL: 4–13%) (Brudno et al., 2018; Berdeja et al., 2019; Cohen et al., 2019; Madduri et al., 2019; Raje et al., 2019) (Jiang et al., 2018) (Figure 5). Hematologic toxic effects were the most frequent treatment-related AEs of grade 3 or higher, including a decreased neutrophil count (70%, 95% CL: 57–81%), anemia (43%, 95% CL: 25–64%), decreased lymphocyte count (43%, 95% CL: 16–75%), and thrombocytopenia (36%, 95% CL: 25–50%).

Subgroup analysis of any-grade CRS by target selection showed that any grade CRS was less frequent in the case of specific BCMA targeting (69.73%) compared to BCMA + CD19/dual BCMA targeting (89.78%) (interaction *p* < 0.05). However, subgroup analysis of grade ≥3 CRS by target selection suggested that, no difference occurred between specific BCMA and BCMA + CD19/dual BCMA targeting. Additional details are shown in Table 6.

**TABLE 6:** The subgroup analysis results of all grade CRS and severe CRS.

Subgroup	No of trails CRS (95% CI) *p* for differences	CRS≥3 (95% CI)	*p* for differences
**Conditions**			
CP	3 0.8625 [0.7690; 0.9367]	0.1627 [0.0359; 0.3453]	
CP/Flu	17 0.7378 [0.5771; 0.8745] 0.1105	0.1028 [0.0387; 0.1858]	0.5905
**CAR-T target**			
BCMA	16 0.6973 [0.5124; 0.8576]	0.0836 [0.0405; 0.1646]	
BCMA + CD19/bispecific BCMA	6 0.8978 [0.8196; 0.9587] 0.0225	0.1641 [0.0380; 0.4935]	0.3979
**Costimulatory domain**			
4-1BB	20 0.7286 [0.5857; 0.8533]	0.0905 [0.0454; 0.1457]	
CD28	4 0.8946 [0.5848; 1.0000] 0.306	0.2311 [0.0000; 0.6907]	0.4317

### 3.5. Sensitivity Analysis

Sensitivity analysis showed that after removal of the largest sample size among all studies, the pooled ORR did not change significantly. Moreover, the results of the meta-analysis were stable (Table 7).

**TABLE 7:** The effect of removing the largest sample size of the study in the sensitivity analysis.

Study No. of patients	Proportion 95%-CI
Total 497	0.8800 [0.8300; 0.9403]
Omitting Zhang et al., 2017, 440	0.8400 [0.8042; 0.8703]

## 4. Discussion

In the last decade, CAR-T therapies have been extensively developed for the advancement of individualized clinical cancer immunotherapy. This meta-analysis, which examined 27 prospective studies involving 497 patients, has demonstrated that CAR-T therapy offered promising outcomes with a tolerable safety profile in RRMM patients.

Our meta-analysis suggests that CAR-T cell therapy could address the negative effects associated with high-risk cytogenetics (≤50% vs. > 50% = 84.21% vs. 82.17%) and exhibited a higher efficacy against MM resistant to previous therapies including IMiDs, PIs, anti-CD38 monoclonal antibody, and ASCT. Notably, patients who did not receive prior ASCT achieved a better response, suggesting that ASCT is an irreplaceable component of RRMM patient treatment.

CAR-T cell-based therapies mechanistically differ from all other MM treatment modalities. CAR-T cells can be optimized to specifically kill tumor cells, or reshape the tumor microenvironment by releasing soluble factors capable of regulating the function of matrix or immune cells (Fujiwara, 2014; Maus et al., 2014; Park et al., 2016). Hence, they represent a powerful tool for targeting multiple constituents of the tumor ecological system (Ye et al., 2018). When stimulated by primary MM cells, anti-BCMA-CAR-transduced T cells produce IFN-*γ* and kill them. In fact, serum from patients receiving BCMA-specific CAR-T cells kill target cells that express BCMA *in vitro* through complement-mediated lysis and antibody-dependent cytotoxicity (Bellucci et al., 2005). Some studies also suggest that earlier CAR-T intervention, at a stage when T cells may be intrinsically “fitter,” may be particularly effective (Kay et al., 2001; Dhodapkar et al., 2003; Suen et al., 2016). Based on these arguments, deciding whether CAR-T therapy should be administered early is challenging, particularly for patients with unfavorable cytogenetics.

Additionally, the efficacy appeared to be independent of conditioning scheme, as the combination of cyclophosphamide/fludarabine (Cy-Flu) appears to produce CAR-T cell dynamics similar to that of cyclophosphamide alone. This differed from the CD19-specific CAR-T cell-based therapy in relapsed/refractory B cell non-Hodgkin’s lymphoma, where Cy/Flu lymphodepletion resulted in higher response rates (50% CR, 72% ORR) compare to those elicited by the Cy-based lymphodepletion without Flu (8% CR, 50% ORR) (Turtle et al., 2016b). Our research demonstrates that the normal expansion and activity of CAR-T cells in MM may not require exhaustive lymphatic depletion, as patients with MM may have intrinsically “fitter” T cell reserves compared to patients with B cell non-Hodgkin’s lymphoma. Therefore, a single CAR-T conditioning protocol may be applied in future patient management.

Previous studies have suggested that specific product features, including the design of engineered costimulation, may impact therapeutic efficacy (Long et al., 2015; Zhao et al., 2015). In contrast, our present study showed that a similar overall response rate (ORR) was elicited by different costimulatory domains (4-1BB, CD28, and CD28 plus OX40), which may indicate that the small patient samples sizes, as well as the diverse differences in study designs, including the inclusion criteria, broad range of efficacious doses, treatment schedule, and lymphodepletion regimen, preclude drawing definitive conclusions. Notably, the production of CAR-T cells depends, to a large extent, on numerous manual, open-process procedures, and cell culture media to reach a clinical therapeutic dosage (Sadelain, 2009; Sadelain et al., 2013). These characteristics may limit the application of this approach to large-scale, multicenter clinical trials. Therefore, studies are needed to streamline and optimize the production process. Moreover, additional steps should be standardized to maximize the process consistency (Roberts et al., 2018).

The initial success of the CD19-targeted CAR-T cell therapy in B-cell malignancy emphasizes that selecting the optimal surface target antigens is critical for efficient CAR-T cell therapeutics. However, first-rank surface antigens remain to be identified in MM. Nevertheless, several alternative antigens have been used in CAR-T cell therapy against MM (Bolli et al., 2014; Tai et al., 2016). In our study, the BCMA, dual BCMA, CD196, CD38, TACI, and FHVH were considered. The results show that LCAR-B38M and combined CD19/BCMA exhibit higher overall response rates and deeper responses compared to specific BCMA. In the design of LCAR-B38M, the antigen recognition portion consists of two camel antibody heavy chains against two BCMA epitopes. This structure may enhance the antigen recognition specifically as well as the affinity of CAR-T cells for antigen, resulting in a stronger anti-MM effect (Shah et al., 2020). In terms of immunophenotype, the dominant clones of most myeloma patients are similar to the most differentiated normal plasma cell subset: CD38 + CD138 + CD19^−^. A few MM clone subsets with poorly differentiated plasma cell phenotypes (CD138lo/– or CD19^+^), or a B cell phenotype (CD138–CD19 + CD20^+^) can also be found in patients. Moreover, according to a clinical trial and *in vitro* study using immunodeficient mice, poorly differentiated components in MM clones are also involved in disease pathogenesis. In addition, CD19 was found to be expressed on only a small proportion of myeloma cells (Bagg et al., 1989; Paiva et al., 2017; Garfall et al., 2018; Nerreter et al., 2019). Hence, the combination of CD19 and BCMA may tackle MM pathogenesis more effectively and result in enhanced anti-tumor effects.

Although our study included some patients without an MRD status reported, the high rate of pooled MRD negativity in patients (81%, 67%–91%) was inspiring. In contrast, a recent study exploring the effects of daratumumab plus pomalidomide–dexamethasone for RRMM showed that 35% and 29% of the patients could be assessed as MRD negative at a threshold of 10^−4^ and 10^−5^ nucleated cells, respectively (Chari et al., 2017). Meanwhile, previous studies showed that the MRD status was one of the most relevant independent prognostic factors in MM. Compared with patients achieving CR who are MRD positive, patients who are MRD negative may have longer overall, and progression-free survival (PFS) (Paiva et al., 2015; Kumar et al., 2016; Munshi et al., 2017). Despite the high response rate, it remains unknown whether CAR-T cells have the potential to induce long-lasting remission in RRMM, as observed with the CD19 CAR-T cells in B-cell malignancy. Longer follow-ups for patients who exhibit a response and are MRD negative will be required to address this question.

CRS was determined to be primarily of grade 1 or 2. The reported incidence of grade 3 or higher with CD19-directed CAR-T cells was 46% with tisagenlecleucel and 13% with axicabtagene ciloleucel (Neelapu et al., 2017; Maude et al., 2018), which is higher than our results (11%). The overall occurrence of grade three or four neurologic toxic events was also low (8%). Generally, the safety profile was tolerable and manageable.

In conclusion, in an era in which numerous novel agents for MM are emerging, CAR-T cells demonstrate a high overall response and a good remission rate in heavily treated patients (Miguel et al., 2013; Lonial et al., 2016; Chen et al., 2018). However, further information regarding the durability of the CAR-T cell-based therapy is needed. Owing to the lack of control groups and the small sample sizes of the examined studies, our results require confirmation by randomized controlled trials. Finally, as continuous development of MM therapeutic agents is underway, the optimization of timing, sequensce, and combination with other therapies will be crucial to obtain adequate responses and substantially increase patient survival (Trudel et al., 2018; Kumar et al., 2019; Parrondo et al., 2020).

## Data Availability Statement

The original contributions presented in the study are included in the article/supplementary material, further inquiries can be directed to the corresponding author.

## Author Contributions

XX collected, analyzed the data, and wrote the article. QH, YO, and WW collected the data, helped in subgroup analysis and prepared the figures and tables. YW and QH designed research, provided the plan and modified the manuscript. All authors read and approved the final manuscript.

## Funding

This research was funded by the National Natural Science Foundation of China (81470327), Sichuan Provincial Academic and Technical Leadership Support Funding Project (2018RZ0137).

## Conflict of Interest

The authors state that the research was performed in the absence of any commercial or financial relationships that could be construed as a potential conflict of interest.
